# Identification of an increased lifetime risk of major adverse cardiovascular events in UK Biobank participants with scoliosis

**DOI:** 10.1136/openhrt-2022-002224

**Published:** 2023-05-03

**Authors:** Valentina Quintero Santofimio, Adam Clement, Declan P O’Regan, James S Ware, Kathryn A McGurk

**Affiliations:** 1 National Heart and Lung Institute, Imperial College London, London, UK; 2 MRC London Institute of Medical Sciences, Imperial College London, London, UK; 3 Royal Brompton and Harefield Hospitals, Guy’s and St. Thomas’ NHS Foundation Trust, London, UK

**Keywords:** Magnetic Resonance Imaging, Heart Failure, Risk Factors

## Abstract

**Background:**

Structural changes caused by spinal curvature may impact the organs within the thoracic cage, including the heart. Cardiac abnormalities in patients with idiopathic scoliosis are often studied post-corrective surgery or secondary to diseases. To investigate cardiac structure, function and outcomes in participants with scoliosis, phenotype and imaging data of the UK Biobank (UKB) adult population cohort were analysed.

**Methods:**

Hospital episode statistics of 502 324 adults were analysed to identify participants with scoliosis. Summary 2D cardiac phenotypes from 39 559 cardiac MRI (CMR) scans were analysed alongside a 3D surface-to-surface (S2S) analysis.

**Results:**

A total of 4095 (0.8%, 1 in 120) UKB participants were identified to have all-cause scoliosis. These participants had an increased lifetime risk of major adverse cardiovascular events (MACEs) (HR=1.45, p<0.001), driven by heart failure (HR=1.58, p<0.001) and atrial fibrillation (HR=1.54, p<0.001). Increased radial and decreased longitudinal peak diastolic strain rates were identified in participants with scoliosis (+0.29, P_adj_ <0.05; −0.25, P_adj_ <0.05; respectively). Cardiac compression of the top and bottom of the heart and decompression of the sides was observed through S2S analysis. Additionally, associations between scoliosis and older age, female sex, heart failure, valve disease, hypercholesterolemia, hypertension and decreased enrolment for CMR were identified.

**Conclusion:**

The spinal curvature observed in participants with scoliosis alters the movement of the heart. The association with increased MACE may have clinical implications for whether to undertake surgical correction. This work identifies, in an adult population, evidence for altered cardiac function and an increased lifetime risk of MACE in participants with scoliosis.

**What is already known on this topic**

- The majority of cardiac studies in patients with scoliosis surround investigations into congenital heart disease. Currently, studies are lacking on the impact of age-related and acquired scoliosis on the functioning of a developmentally normal heart.

**What this study adds**

- This study is the first to investigate an interplay between scoliosis and cardiac manifestations in adults of the UK Biobank. We identified an increased lifetime risk of major adverse cardiovascular events, driven by heart failure and atrial fibrillation, and altered radial and longitudinal peak diastolic strain rates in participants with scoliosis.

**How this study might affect research, practice or policy**

- Scoliosis may be an important modifier of cardiac strain and lifetime risk of major adverse cardiovascular event in the adult population. This has clinical implications for the consideration of undertaking scoliosis treatment surgery to alleviate the cardiac burden.

## Introduction

Scoliosis is the lateral curvature of the spine with a Cobb angle >10°, primarily diagnosed in adolescents.^1^ There are multiple aetiologies of scoliosis including neuromuscular, congenital, syndromic or secondary to other diseases such as muscular dystrophy or Friedreich’s ataxia.^2^ However, the most common type is idiopathic scoliosis, with a prevalence of 8% in adults aged over 40 years old.^3–5^ In later life, scoliosis can result from skeletal muscle diseases such as sarcopenia. Sarcopenia is the loss of muscle mass associated with age that may cause imbalance and alteration on the supportive muscles of the spine, contributing to the progression of degenerative scoliosis in elderly patients.^6 7^ Degenerative scoliosis is observed in 68% of adults aged over 60 years old, as the joints and disks of the spine begin to deteriorate.^7^ Osteopenia, loss of bone density, is more frequently observed in women and contributes to the severity of the curvature of the spine.^8^ Furthermore, the coexistence of congenital heart disease (CHD) and spinal curvature is found in up to 12% of infant and juvenile patients with scoliosis presenting with CHD.^9 10^ This is likely due to shared developmental aetiology, whereas studies are lacking on the impact of age-related scoliosis on the functioning of a developmentally normal heart.

In addition to impacting physical day-to-day activities, structural disruption of the thoracic cage (pectus deformity) can influence the organs within, such as the heart.^11^ Pectus deformities lead to the displacement of the heart towards the left side of the body which can result in right-sided spinal curvatures as the beating heart pushes the thoracic vertebrae to the right.^7 11^ Furthermore, the degree of curvature of the spine can greatly increase the risk of restrictive lung disease, which in conjunction with intrathoracic organ displacement, increases the risk of comorbidities with high mortality rates, such as right heart failure.^3 12^

The impact of scoliosis on adult cardiac function has not been extensively studied and the relationship between scoliosis and non-congenital cardiac manifestations is not well characterised. In the UK Biobank (UKB) adult population cohort, we explored whether all-cause scoliosis has an impact on or relationship with cardiac phenotypes of the matured adult heart. We identify altered radial and longitudinal peak diastolic strain rates and an increased lifetime risk of major adverse cardiovascular events (MACEs) in participants with scoliosis in the UKB. In addition, we observed associations between scoliosis and older age, female sex, heart failure, valve disease, hypercholesterolemia, diagnosis of hypertension and decreased enrolment for cardiac MRI (CMR).

## Methods

### The UKB population cohort

The UKB recruited over 500 000 participants aged 40–69 years across the UK between 2006 and 2010 (National Research Ethics Service, 11/NW/0382, 21/NW/0157).^13^ This project was conducted under the UKB applications 47602 and 40616. All participants provided written informed consent.^14^

### UKB codes for identification of scoliosis

A diagnosis of scoliosis was identified for UKB participants through the first occurrence of scoliosis trait (M41*), a composite trait of the first reported date derived by the UKB that incorporates data from primary care, hospital inpatient admissions, death records and self-reported medical conditions (table 1).

**Table 1:** Codes used for identification of all-cause scoliosis

Disease/phenotype	Participants (n=4095)	Coding system
Self-reported scoliosis	468 (11.4%)	SR: 1535
Kyphoscoliosis and scoliosis	5 (0.1%)	ICD9: 7373, 75 420
Congenital postural scoliosis	0 (%)	
Congenital scoliosis	10 (0.2%)	ICD10: Q763, M965, M41, M410, M4100–109, M411, M4110–119, M412, M4120–129, M413, M410–139, M414, M1410–149, M415, M1450–159, M418, M4180–189, M419, M4190–199
Post-radiation scoliosis	10 (0.2%)	
Childhood scoliosis (infantile and juvenile)	23 (0.6%)	
Other idiopathic scoliosis	56 (1.4%)	
Thoracogenic scoliosis	24 (0.6%)	
Neuromuscular scoliosis	22 (0.6%)	
Secondary scoliosis	79 (2%)	
Other forms of scoliosis	226 (5.5%)	
Unspecified scoliosis	2685 (65.5%)	
Unspecified scoliosis	487 (11.9%)	GP-derived

The HES data are coded using the International statistical Classification of Disease (ICD) codes, versions 9 and 10. The self-reported data (SR) have a UKB-specific coding system.

GP, general practitioner; ICD9, summary diagnoses (main and secondary); ICD10, underlying primary cause of death, contributory cause of death, and external cause of death, summary diagnoses (main and secondary); SR, non-cancer illness code, self-reported data.

### CMR data

Among all UKB participants, 39 559 participants had CMR data available.^15^ Imaging was performed using a 1.5 Tesla machine (MAGNETOM Aera, Siemens Healthcare, Erlangen, Germany),^15^ and 2D summary CMR traits were analysed for an association with scoliosis. This includes left ventricular ejection fraction, left ventricular end systolic volume, left ventricular end diastolic volume and measures of cardiac strain: Eulerian radial strain, Eulerian longitudinal strain, Eulerian circumferential strain, radial peak diastolic strain rate (PDSR_rr_) and longitudinal peak diastolic strain rate (PDSR_ll_). All CMR traits were adjusted for age at the time of imaging, sex, white British ancestry, systolic blood pressure (SBP) and body surface area (BSA).

### Surface to surface analysis

A mass univariate regression was used to explore associations between the three-dimensional (3D) mesh-derived phenotype and scoliosis.^16–19^ The underlying principle of this approach is the implementation of a linear regression at each vertex of the 3D atlas to derive a regression coefficient associated with the variable of interest, which results in a map of beta-coefficients showing the strength and direction of these associations. The analysis was adjusted for age at the time of imaging, sex, white British ancestry, BSA, diastolic blood pressure (DBP) and SBP.

### Lifetime risk and survival analyses

Lifetime risk of disease, from birth month to January 2021, was assessed using the first occurrence of scoliosis trait (M41*). The first occurrence of health outcomes summary data in the UKB is reported using ICD10 codes.^13^ The fields analysed for the MACEs composite trait were as follows: cardiac arrest, I46*; atrial fibrillation and flutter/arrhythmia, I48* and I49*; heart failure, I50* and stroke, I64*, as previously published.^20^ The survival analysis was conducted using MACE and death as the primary outcome. *Survival* and *survminer* R packages were used to estimate HRs.

### Statistical analysis

R programming language (V.3.6.0) and RStudio software (V.1.3.1073) were used for analyses. Categorical variables were assessed using χ^2^ test or Fisher’s exact test and expressed as percentages. Continuous variables were assessed using Student’s t-test and expressed as mean±SD. All p values were adjusted using Bonferroni correction for multiple comparisons where P_adj_ <0.05 was deemed significant.

In the association of disease analysis, all variables were adjusted for age at recruitment (UKB ID: 21022-0.0) and genetic data-derived sex (UKB ID: 22001-0.0) using a multiple linear regression model. A separate multiple linear regression model for CMR traits was used to adjust for covariates at the time of imaging: age, sex, white British ancestry, SBP and BSA.

## Results

### Prevalence of scoliosis in the UKB

The prevalence of all-cause scoliosis in 502 324 participants of the UKB was 1 in 120 (n=4095; 0.8%). Of the 4095 participants with scoliosis, 1489 (37%) were diagnosed with scoliosis prior to recruitment. Of the total participants with scoliosis, 10 (0.2%) reported congenital scoliosis, 23 (0.6%) reported childhood scoliosis (infantile and juvenile) and the rest reported scoliosis due to other causes later in life. The most common scoliosis subtype was unspecified scoliosis reported by 2685 (65.6%) participants (table 1).

**Figure 1:**
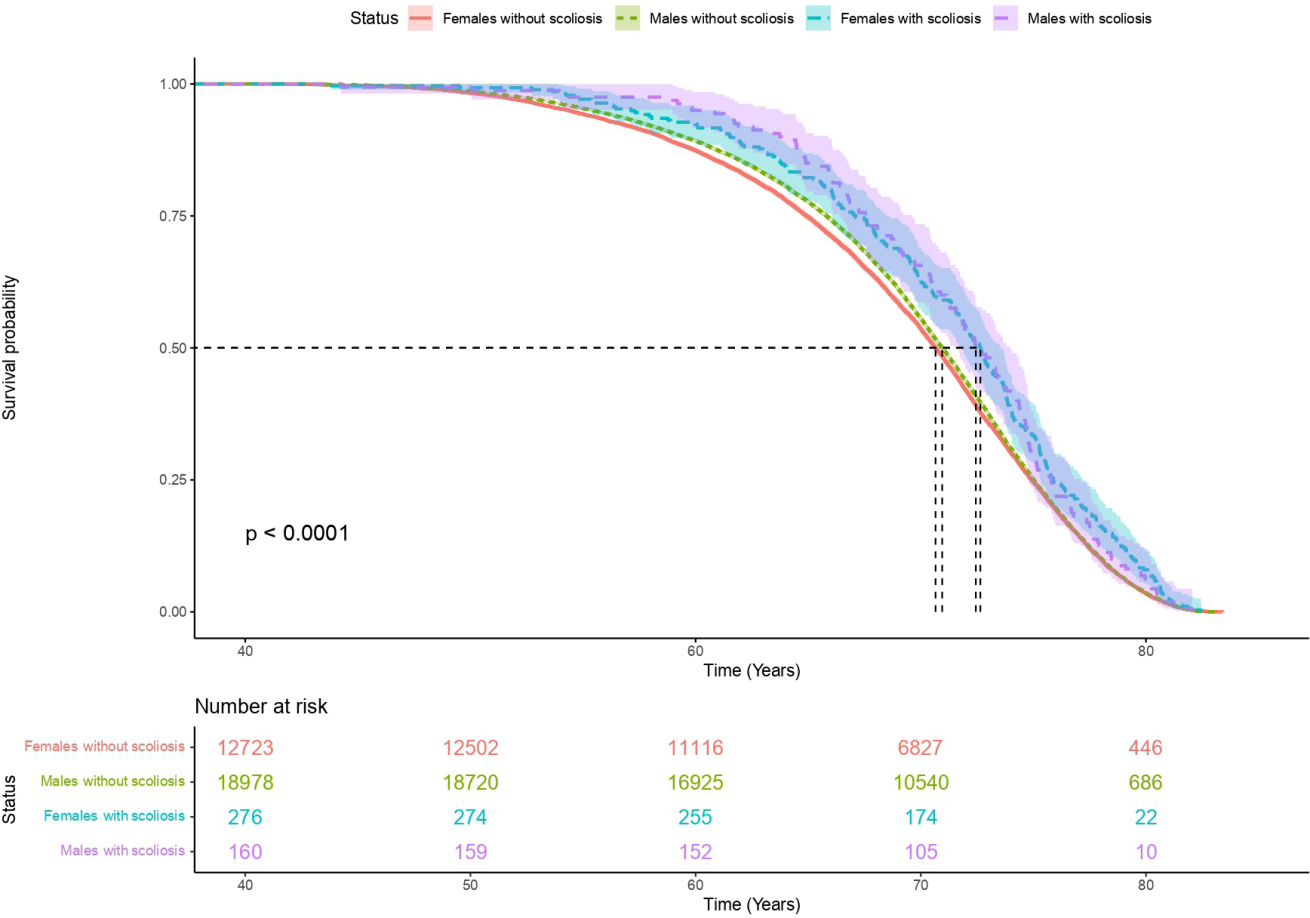
Scoliosis is associated with longer life compared with the rest of the UKB cohort, adjusted for sex. Years of life is shown on the x-axis from date of birth. Counts of UKB participants are shown in the box depicting number at risk. The median survival time (a survival probability of 0.5) was significantly increased in participants with scoliosis, regardless of sex. Median survival time for females with scoliosis versus females without scoliosis (2 years difference); median survival time for males with scoliosis versus males without scoliosis (1.5 years difference). UKB, UK Biobank.

Participants with scoliosis were significantly older than the rest of the population (59.4 years old±7.58; mean age 56.5 years old±8.10; P_adj_ <0.01) and were more female (69% women in scoliosis cohort; 55% women in the rest of the UKB population; P_adj_ <0.01). Lifetime risk analysis showed that participants with scoliosis had significantly longer lives when compared with the rest of the population, regardless of sex (figure 1).

We observed a significantly increased burden of heart failure (+4%, P_adj_ <0.001), valve disease (+1%, P_adj_ <0.001), hypercholesterolemia (+7%, P_adj_ <0.001) and diagnosis of hypertension (+13%, P_adj_ <0.001) in participants with scoliosis, adjusted for age and sex (table 2). Although diagnosis of hypertension was significantly increased, no significant differences were found with SBP or DBP, which may be due to the correction of diagnosed hypertension through medication. Additionally, the proportion of participants with scoliosis and CMR available was significantly decreased (−3%, P_adj_ <0.001) compared with the rest of the population.

**Table 2:** Summary statistics of participants with scoliosis compared with the rest of the UK Biobank population cohort

Characteristic	General population (n=502 324)	Scoliosis carriers (n=4095)	P_adj_
Age at recruitment	56.50±8.10	59.40±7.58	<0.001
White British	406 131 (81%)	3344 (82%)	NS
Sex, female	261 862 (55%)	2845 (69%)	<0.001
BMI	27.40±4.80	27.00±5.16	NS
Auto SBP	140.00±19.60	141.00±20.00	NS
Auto DBP	82.20±10.70	81.50±10.80	NS
Diabetes	27 771 (5%)	212 (5%)	NS
Hypercholesterolaemia	92 824 (18%)	1022 (25%)	<0.001
Hypertension	167 817 (33%)	1902 (46%)	<0.001
Included in UKB CMR cohort	39 319 (8%)	224 (5%)	<0.001
Heart failure	10 504 (2%)	237 (6%)	<0.001
Any cardiomyopathy	3602 (0.7%)	49 (2%)	NS
Valve disease	5145 (1%)	96 (2%)	<0.001

Data are presented as mean±SD or count (%); all variables were adjusted for age and sex to assess significance. White British ancestry was genetically inferred. P_adj_, p value adjusted for multiple comparisons via Bonferroni correction; significant, P_adj_ <0.05; NS, not significant, P_adj_ >0.05.

BMI, body mass index; CM, cardiomyopathy; CMR, cardiac MRI; DBP, diastolic blood pressure; SBP, systolic blood pressure.

### CMR analysis of participants with scoliosis identifies altered cardiac diastolic strain

2D summary CMR traits were available for 39 559 participants.^15^ Two hundred and twenty-four participants with scoliosis and CMR available had significantly increased radial PDSR (P_adj_ <0.05) and decreased longitudinal PDSR (P_adj_ <0.05) compared to participants without a diagnosis of scoliosis (table 3). Adequate diastolic function is essential during ventricular filling and maintenance of optimum stroke volume. PDSR is a diastolic function trait, that has been previously associated with MACEs, increased mortality, increased blood pressure, and altered left atrial function.^21^ For example, decreased PDSR_rr_ corresponds to stiffer ventricle, impairing relaxation and increasing the risk of heart failure.^21^ No additional significant associations were found with other CMR measures available for analysis and no association was observed between scoliosis and diabetes (table 2).

**Table 3:** Altered cardiac PDSR in participants of the UKB with scoliosis

CMR trait	General population (n=38 319)	Scoliosis (n=224)	P_adj_
Maximum WT	9.40±1.62	9.36±1.70	NS
LVEDV	148.00±33.90	136.00±32.80	NS
LVEF	59.50±6.16	59.80±6.03	NS
LVM	86.0±22.30	78.00±20.70	NS
Ecc Global	−22.33±3.35	−22.10±3.77	NS
Err Global	45.00±8.31	45.20±8.73	NS
Ell Global	−18.50±2.76	−17.90±2.44	NS
PDSR_ll_	1.66±0.61	1.45±0.58	<0.05
PDSR_rr_	−5.64±2.09	−5.08±2.13	<0.05

Mean and SD for CMR-derived summary measures are described for the general UKB population and participants with all-cause scoliosis. The p value was adjusted for multiple comparisons of CMR traits.

CMR, cardiac MRI; Ecc, Eulerian circumferential strain; Ell, Eulerian longitudinal strain; Err, Eulerian radial strain; LVEDV, left ventricular end-diastolic volume; LVEF, left ventricular ejection fraction; LVM, left ventricular mass; PDSR_ll_, longitudinal peak diastolic strain rate; PDSR_rr_, radial peak diastolic strain rate; UKB, UK Biobank; WT, wall thickness.

### Surface to surface analysis of participants with scoliosis shows increased cardiac compression

**Figure 2:**
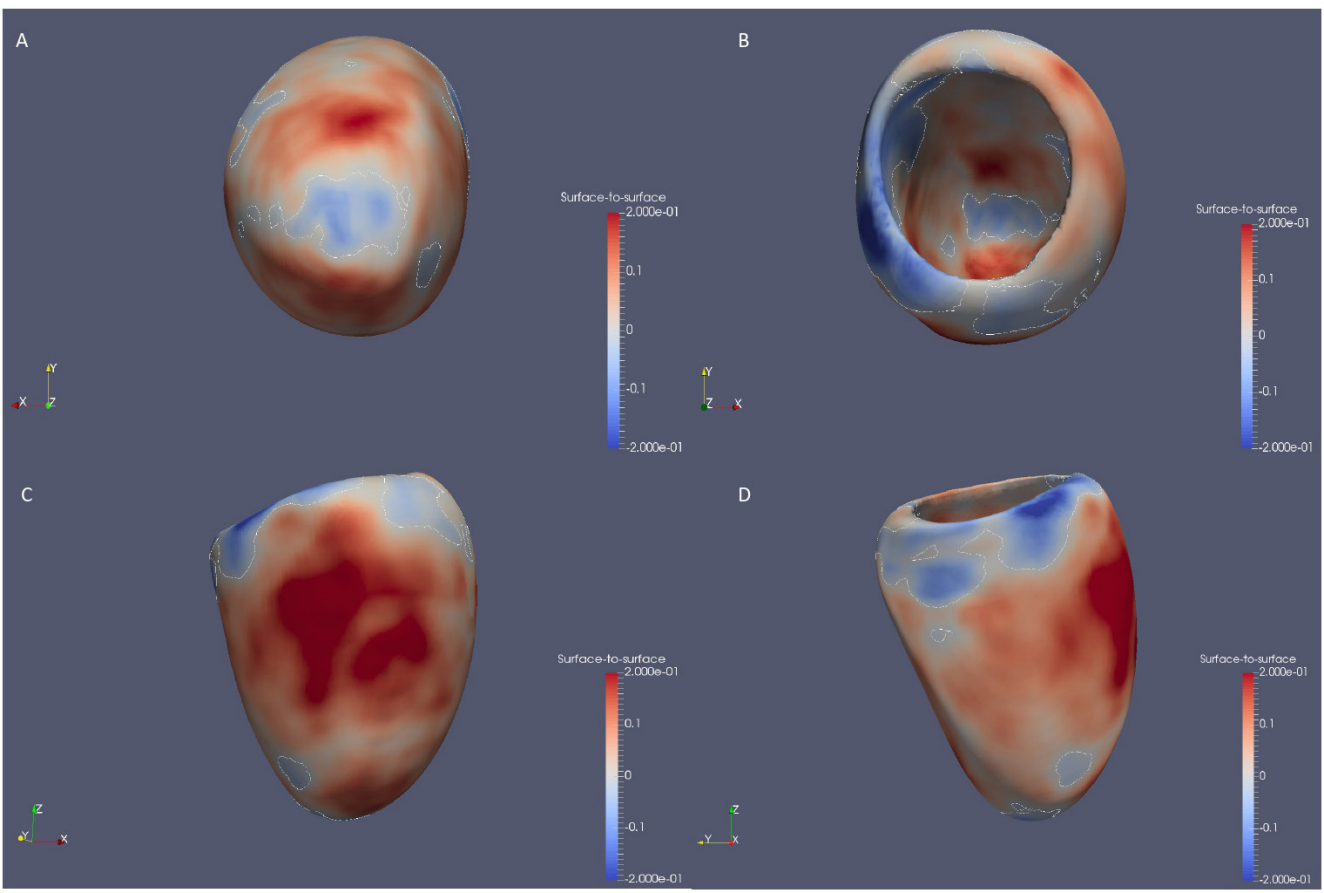
Surface to surface analysis suggests a compressed heart in participants with scoliosis. 3D models of left ventricle geometry with standardised beta-coefficients which show the association between scoliosis and regional surface-to-surface distance. Blue, increased inward pressure; red, increased outwards pressure. 3D, three-dimensional.

A 3D surface-to-surface analysis was performed on 21 088 participants of the cohort. The 3D cardiac modelling of patients with scoliosis showed increased strain at the top and bottom of the heart (figure 2). Radial cardiac decompression (sides of the heart) was also observed in participants with scoliosis. However, these were not significant when adjusting for the number of comparisons and covariates.

### Lifetime risk of MACEs is increased in participants with scoliosis

**Figure 3:**
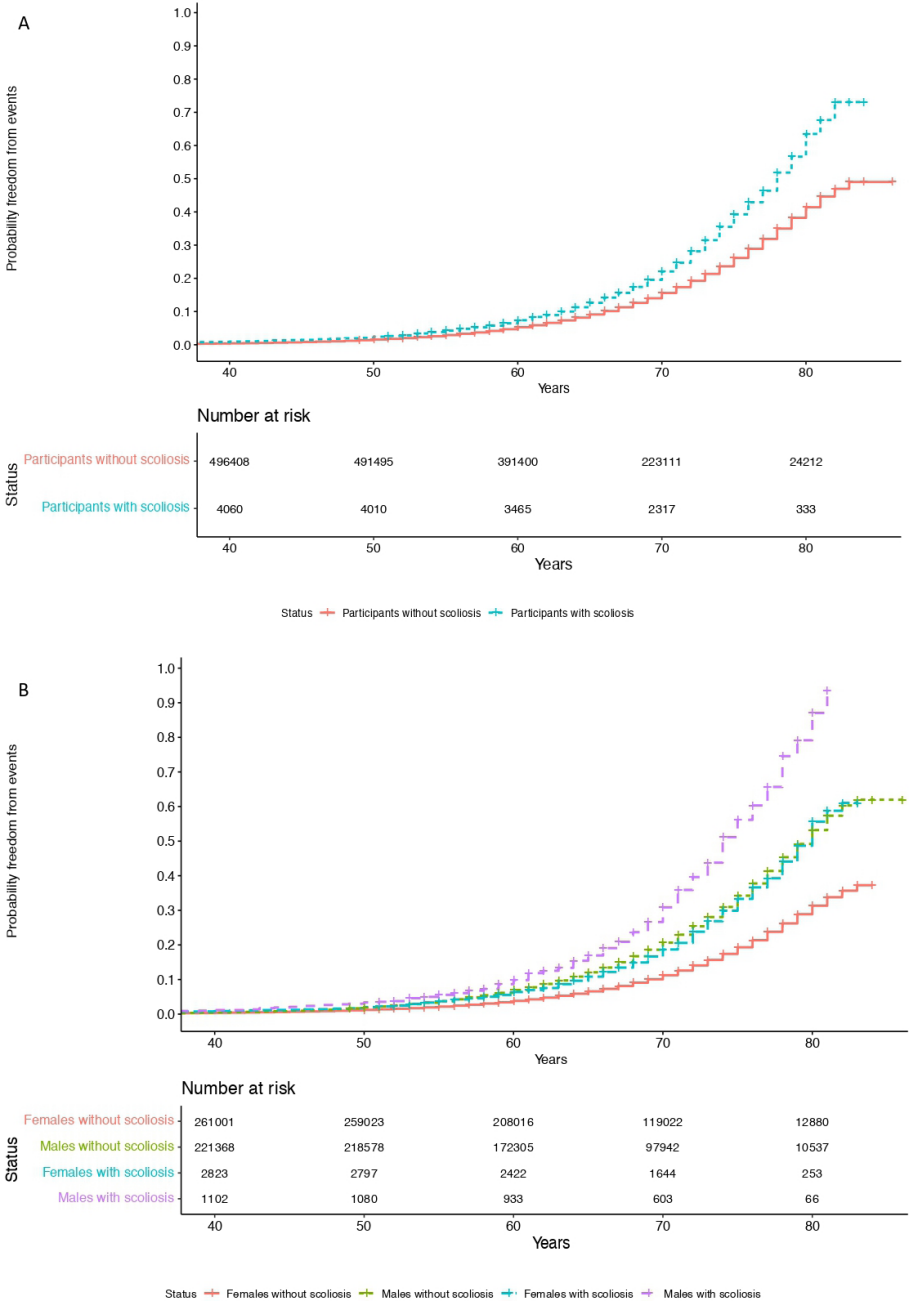
Increased lifetime risk of MACEs in UKB participants with scoliosis. Cumulative incidence curves depict (A) an increase in lifetime risk of MACE in participants with scoliosis over time (HR=1.45, p<0.001) and (B) stratified by sex (HR=1.63, p<0.001). MACEs, major adverse cardiovascular events; UKB, UK Biobank.

A significantly increased lifetime risk of MACEs was observed for UKB participants with scoliosis (figure 3; HR=1.45, p<0.001; stratified by sex, HR=1.63, p<0.001), mainly driven by heart failure (HR=1.58, p<0.001) and atrial fibrillation (HR=1.54, p<0.001). The probability of MACE doubled in men into older age (from 60 years of age). This may be caused through the altered cardiac diastolic strain rates observed in participants with scoliosis. However, we emphasise caution regarding the causality of this association of scoliosis and MACEs at this stage.

## Discussion

### Participants with scoliosis have an increased lifetime risk of MACEs

To the best of our knowledge, this is the first study to identify an increased lifetime risk of MACEs, through atrial fibrillation and heart failure, in participants with scoliosis. This may be through the identified, significantly increased PDSR_rr_ and decreased PDSR_ll_. The abnormal curvature of the spine can increase mechanical constraint on the heart which may result in diastolic dysfunction^22^ and the severity of the spinal deformity has been shown to aggravate ventricular and right atrial pressure.^3^

It is unlikely that the observed heart failure is due to blood pressure abnormalities as no significant differences were found in measured SBP and DBP between participants with scoliosis and the rest of the population. However, it is possible that pulmonary dysfunction may be eliciting cardiac dysfunction alongside atrial fibrillation, in patients with advanced scoliosis.^3 11 23^ These findings suggest that early medical intervention in patients with scoliosis through surgery may decrease the risk of a future MACEs, however, future validation of this finding is needed in a scoliosis case cohort. It may be possible that scoliosis develops secondary to other diseases that could also increase the risk of MACEs. Future genetic analyses would be beneficial to assess causality through Mendelian randomisation techniques. Likewise, future studies are required to determine whether the cardiovascular changes observed are reversible with scoliosis treatment surgery.

A previous study assessed 201 patients with scoliosis for cardiopulmonary changes following corrective scoliosis surgeries and suggested that untreated scoliosis can result in pulmonary dysfunction and subsequently lead to right heart failure, increasing mortality.^3^ Postoperative normalisation of cardiac measures was observed in this study, highlighting the potential benefits of surgical correction of scoliosis on cardiac function.^3^ Additionally, a long-term follow-up study showed increased risk of pulmonary limitations such as shortness of breath in patients with untreated scoliosis, aggravated by spinal curvature.^24^

It is challenging to accurately assess the cardiac function of patients with scoliosis, due to the heart being displaced^22^ and participants with scoliosis in the UKB had significantly less CMR scans available, possibly due to lack of comfort for prolonged periods of time in MRI machines. Adaptations may be required to allow patients with scoliosis to undergo MRI scans more comfortably and to further assess any alteration in cardiac function in patients with scoliosis.

### Participants with scoliosis have altered cardiac diastolic strain rates

In the UKB, participants with scoliosis showed increased pressure at the top and bottom of the heart, as well as elongation of the sides of the heart. These results concur with the 2D-derived CMR findings of significantly increased PDSR_rr_ and decreased PDSR_ll_. A reduced PDSR_ll_ has been previously associated with reduced left atrial function.^21^ No significant associations were found with the other studied CMR traits, suggesting that this compression does not alter blood flow through the heart. This altered cardiac strain may be explained by the deformity of the thoracic cage in scoliosis limiting cardiac diastolic movement. The mechanical abnormalities of the thoracic spine as well as the impact on the pulmonary system may be the primary cause of the heart involvement.^25 26^ Secondary involvement via altered pulmonary haemodynamics may be possible, where spinal curvature impacts pulmonary pressures leading to pulmonary hypertension. Likewise, direct compression of the myocardium could occur in conjunction with pulmonary involvement. These events can contribute to the development of cardiac consequences in patients with scoliosis.^27^ It would be beneficial to include cardiac follow-ups in patients with scoliosis to observe any cardiac alterations as scoliosis progresses, ensuring early intervention.

### Prevalence of scoliosis in the UKB

We report a prevalence of scoliosis of 0.8% in the UKB population cohort. Previous literature has reported a scoliosis prevalence of 8% in adult volunteers aged over 40 years old.^5^ Patients included in the previous clinical study were recruited on evaluation of bone mineral density and thus were likely at increased risk of scoliosis compared to the UKB population cohort. Additionally, this discrepancy may be due to the limitations of the UKB cohort (see the Limitations section).

Participants with scoliosis in the UKB are more elderly compared with the rest of the population, regardless of sex. Scoliosis occurs alongside other diseases such as osteoporosis or degenerative spine disorders with increasing age.^5^

In the UKB cohort, scoliosis is more commonly found in women than men (2.5:1), which agrees with a previous scoliosis case cohort study of adolescents and adults that reported a ratio of scoliosis in women to men as 2:1.^28^ Although not fully understood, there are different theories linking the higher prevalence of scoliosis in women, including the implication of the autonomic nervous system in skeletal growth and/or puberty through leptin hormone, which has been found decreased in female patients with idiopathic scoliosis.^1 29 30^ Analyses to determine causality would aid our current understanding of the mechanisms behind scoliosis. Furthermore, it has been reported that approximately 30% of women (n=324) suffer from systemic osteopenia which strongly contributes to the progression of spinal curvature in adolescent females following skeletal maturity.^8^

### Limitations

The participants in the UKB cohort were recruited at 40–69 years of age and most participants are of European ancestry. Selection bias for inclusion in CMR subcohort may have excluded participants with more severe scoliosis from the study. In addition, discrimination of scoliosis aetiology was mostly unspecified and information was not available on measures of scoliosis severity.

## Conclusions

This work describes for the first time in an adult population, evidence for altered cardiac function in adult participants with scoliosis. We identified altered diastolic strain, increased lifetime risk of MACE driven by heart failure and atrial fibrillation, and observed cardiac compression in the UKB participants with scoliosis. Further research is required to follow-up the role of scoliosis in cardiac manifestations in a clinical setting.

10.1136/openhrt-2022-002224.supp1 Supplementary data

## Data Availability

UK Biobank (https://www.ukbiobank.ac.uk/) population reference datasets are publicly available. Analysis code is available on GitHub (https://github.com/ImperialCardioGenetics/Scoliosis).
