# Supplementary material for: Identification of an increased lifetime risk of major adverse cardiovascular events in UK Biobank participants with scoliosis
Source: Open Heart. 2023 May 3;10(1):e002224. doi: 10.1136/openhrt-2022-002224 (PMC10163590; doi:10.1136/openhrt-2022-002224)
Supplement: Supplementary data [file openhrt-2022-002224supp001.pdf]

Exploration of the impact of scoliosis on the heart in the UK Biobank

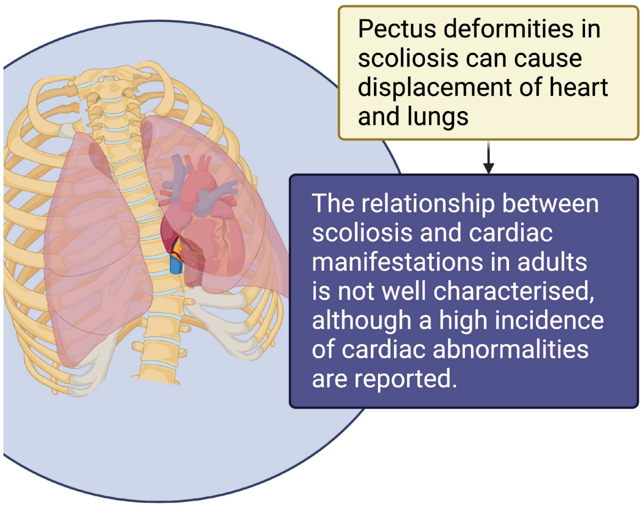

Pectus deformities in scoliosis can cause displacement of heart and lungs

The relationship between scoliosis and cardiac manifestations in adults is not well characterised, although a high incidence of cardiac abnormalities are reported.

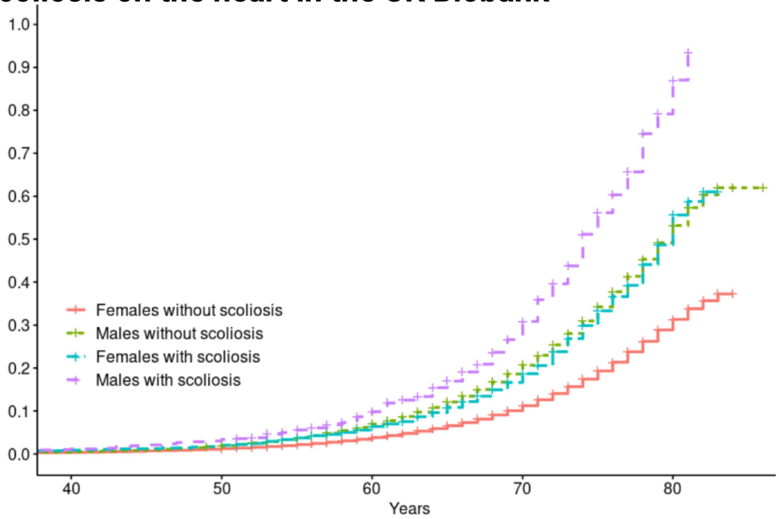

4,095 (1 in 120) participants have scoliosis in the UK Biobank with:
Increased risk of major adverse cardiovascular events (HR=1.63)
Mainly driven by heart failure (HR=1.58) and atrial fibrillation (HR=1.54)

Cardiac magnetic resonance imaging
Diastole

No spinal curvature | Scoliosis

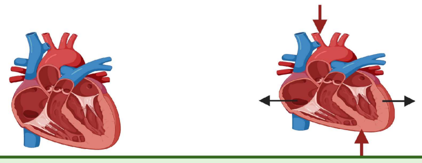

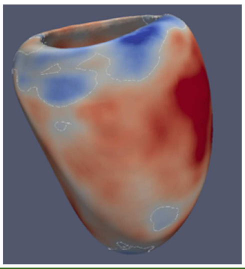

Surface to surface analysis

Identification of increased cardiac compression on the top and bottom of the heart, and radial cardiac decompression (sides of the heart) in scoliosis patients.

Conclusion:
This work identifies, in an adult population, the evidence for altered cardiac function and increased lifetime risk of MACE in participants with scoliosis.
